# Synthesis and DFT Calculations of Novel Vanillin-Chalcones and Their 3-Aryl-5-(4-(2-(dimethylamino)-ethoxy)-3-methoxyphenyl)-4,5-dihydro-1*H*-pyrazole-1-carbaldehyde Derivatives as Antifungal Agents

**DOI:** 10.3390/molecules22091476

**Published:** 2017-09-05

**Authors:** Luis Alberto Illicachi, Joel José Montalvo-Acosta, Alberto Insuasty, Jairo Quiroga, Rodrigo Abonia, Maximiliano Sortino, Susana Zacchino, Braulio Insuasty

**Affiliations:** 1 Grupo de Investigación de Compuestos Heterocíclicos, Departamento de Química, Universidad del Valle, A. A. 25360 Cali, Colombia; luis.illicachi@correounivalle.edu.co (L.A.I.); Jairo.quiroga@correounivalle.edu.co (J.Q.); rodrigo.abonia@correounivalle.edu.co (R.A.); 2 Laboratoire d’Ingénierie des Fonctions Moléculaires, Institut de Science et d’Ingénierie Supramoléculaires, UMR 7006, CNRS, F-6700 Strasbourg, France; montalvo161@gmail.com; 3 Departamento de Química y Biología, Universidad del Norte, Km 5 vía Puerto Colombia, Barranquilla 081007, Colombia; binsuasty@uninorte.edu.co; 4 Área Farmacognosia, Facultad de Ciencias Bioquímicas y Farmacéuticas, Universidad Nacional del Rosario, Suipacha 531, 2000 Rosario, Argentina; msortino@fbioyf.unr.edu.ar (M.S.); szaabgil@citynet.net.ar (S.Z.)

**Keywords:** antifungal activity, cyclocondensation reaction, chalcones, *N*-aryl-2-pyrazolines, DFT calculations

## Abstract

Novel (*E*)-1-(aryl)-3-(4-(2-(dimethylamino)ethoxy)-3-methoxyphenyl) prop-2-en-1-ones **4** were synthesized by a Claisen-Schmidt reaction of 4-(2-(dimethylamino)ethoxy)-3-methoxy-benzaldehyde (**2**) with several acetophenone derivatives **3**. Subsequently, cyclocondensation reactions of chalcones **4** with hydrazine hydrate afforded the new racemic 3-aryl-5-(4-(2-(dimethylamino)ethoxy)-3-methoxyphenyl)-4,5-dihydro-1*H*-pyrazole-1-carbaldehydes **5** when the reaction was carried out in formic acid. The antifungal activity of both series of compounds against eight fungal species was determined. In general, chalcone derivatives **4** showed better activities than pyrazolines **5** against all tested fungi. None of the compounds **4a**–**g** and **5a**–**g** showed activity against the three *Aspergillus* spp. In contrast, most of the compounds **4** showed moderate to high activities against three dermatophytes (MICs 31.25–62.5 µg/mL), being **4a** followed by **4c** the most active structures. Interestingly, **4a** and **4c** possess fungicidal rather than fungistatic activities, with MFC values between 31.25 and 62.5 μg/mL. The comparison of the percentages of inhibition of *C. neoformans* by the most active compounds **4**, allowed us to know the role played by the different substituents of the chalcones’ A-ring. Also the most anti-cryptococcal compounds **4a**–**c** and **4g**, were tested in a second panel of five clinical *C. neoformans* strains in order to have an overview of their inhibition capacity not only of standardized but also of clinical *C. neoformans* strains. DFT calculations showed that the electrophilicity is the main electronic property to explain the differences in antifungal activities for the synthesized chalcones and pyrazolines compounds. Furthermore, a quantitative reactivity analysis showed that electron-withdrawing substituted chalcones presented the higher electrophilic character and hence, the greater antifungal activities among compounds of series **4**.

## 1. Introduction

The increase in fungal infections during recent years is strongly related the growing number of immunocompromised patients [1,2]. Most prevalent mycoses are classified either as superficial or systemic. Superficial infections are caused by fungi of *Candida* genus or by dermatophytes, a type of fungi that comprises species of the *Microsporum* and *Trichophyton* genera [3,4], while systemic mycoses are mainly produced by *Candida* or *Aspergillus* spp. and *Cryptococcus neoformans* [5]. Although there are different antifungal drugs for the treatment of fungal infections, their management is markedly limited by problems of toxicity, resistance and effectiveness profiles [2,6]. There is, therefore, a clear need for the discovery of new structures with antifungal properties, which could lead to the development of new drugs for the treatment of fungal infections.

The chalcone and pyrazoline moieties are important classes of compounds widely used as key building blocks for biologically active compounds and they are considered promising candidates for antifungal drugs. Chalcones have been extensively studied for their broad spectrum of activities as anti-inflammatory [7], antifungal [8,9], antibacterial [10], antioxidant [11], antimalarial [12,13] and antitumor [14,15] agents. One of our previous studies demonstrated that the mode of antifungal action of certain chalcones is related to the inhibition of the synthesis of the fungal cell-wall polymers such as (1,3)β-d-glucan synthase. From a therapeutic standpoint chalcones can inhibit glutathione-*S*-transferases (GSTs), enzymes that have been demonstrated to be involved in drug resistance [16,17]. In addition, previous works have demonstrated that the antifungal activity of chalcones depended on their substitution pattern as well as on the yeast genotype and strain cell density. Related to substitution patterns, some authors have found that EWG groups at the *para*-position increase the electron deficiency at C-*β*, transforming it into an attractive electrophilic centre for thiol attack, but the EDG groups hamper this reaction decreasing the antifungal activity. The same effect was seen when EWG groups were at the *ortho*-position due to steric effects [18,19].

Similarly, the substituted 2-pyrazoline moiety represents a structural component of significant interest in the medicinal chemistry field, due to its prominent pharmacological effects, such as antimicrobial [20], antifungal [21], anti-inflammatory [22], antimalarial [23], and anticancer [24] activities.

Previous studies have also shown that the use of vanillin compounds as starting materials in organic synthesis led to the formation of several vanillin-derivatives with a wide spectrum of biological activities [10,25,26,27].

On the other hand, density functional theory (DFT), after its inception few decades ago, has been recognized as a powerful tool to provide theoretical insights into chemical reactivity and how this influences the biological properties of drug-like compounds [28]. Thus, DFT calculations have been applied to generate quantum mechanical descriptors used in quantitative structure-activity relationship (QSAR) studies in order to explain a broad spectrum of biological properties of pharmacologically-relevant scaffolds such as the antioxidant activity in flavonoids [29] or the antimalarial activity in quinolones derivatives [30].

In this paper we describe the synthesis of new chalcones and pyrazoline derivatives, containing the vanillin moiety in their structures, and the evaluation of their antifungal activity against a panel of clinically important fungi, including yeasts, filamentous fungi as well as dermatophytes. In addition, a chemical reactivity analysis based on DFT calculations is performed to obtain a major understanding of the antifungal activities for these synthesized chalcones and pyrazolines.

## 2. Results

### 2.1. Chemistry

**Scheme 1:**
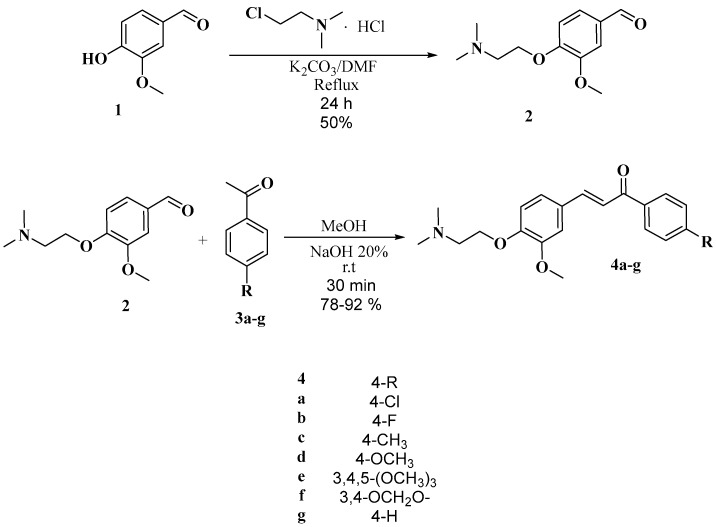
Synthesis of new vanillin chalcones **4a**–**g**.

In order to obtain chalcone derivatives containing the vanillin moiety, the precursor 4-[2-(dimethylamino)ethoxy]-3-methoxybenzaldehyde (**2**) was synthesized by alkylation of the OH group of vanillin (**1**) with the **2**-chloro-*N*,*N*-dimetylamine hydrochloride and K_2_CO_3_. DMF was used as the solvent of the reaction, which was carried out under reflux (Scheme 1). Compound **2** was characterized by using FT-IR, ^1^H-NMR and MS techniques that allowed to elucidate its structure by comparing the data obtained with those reported in the literature for the same compound [31]. Subsequently, the condensation reaction of Claisen-Schmidt between the aldehyde **2** and different acetophenones **3a**–**g**, was carried out affording the corresponding chalcones **4a**–**g** (Scheme 1).

The structural elucidation of compounds **4a**–**g** was carried out by means of FT-IR, ^1^H-NMR, ^13^C-NMR and MS techniques. The FT-IR spectra showed main absorption bands at 1654 (C=O), 1596 (C=C), 1207 and 1253 (C-N) and 1020 (C-O-C) cm^−1^. In the ^1^H-NMR spectra of compounds **4a**–**g** the H-*β* and H-*α* protons appeared each one as a doublet at *δ* = 7.75–7.73 and *δ* = 7.32–7.39 ppm respectively, with a coupling constant between them of ^3^*J* = 15.6 Hz, which agrees with a *trans* configuration. Analysis of ^13^C, DEPT-135 and 2D-heteronuclear NMR spectra (HSQC and HMBC) provided the final structure elucidation of compounds **4a**–**g** (see Experimental Section). Thus, for the compounds **4a**–**g** the C-*β* and C-*α* signals appeared at *δ* = 138.4–148.2 and *δ* = 120.1–123.6 ppm respectively, while the C=O appeared at *δ* = 188.9–190.7 ppm. Finally, the mass spectra of compounds **4a**–**g** showed well-defined molecular ions in all cases.

**Scheme 2:**
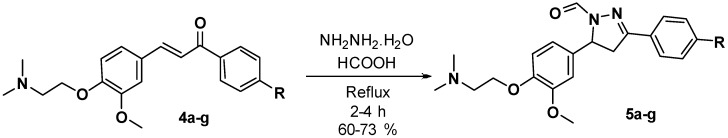
Synthesis of new *N*-formyl pyrazolines **5a**–**g** derivatives of the vanillin chalcones.

In a second step, 1,3-dielectrophile cyclization reactions of chalcones **4a**–**g** were carried out, employing a three-part methodology, in which the chalcones were mixed with hydrazine hydrate and formic acid. The results suggest that the formic acid acted not only as solvent but also as a formylating agent (Scheme 2).

**Figure 1:**
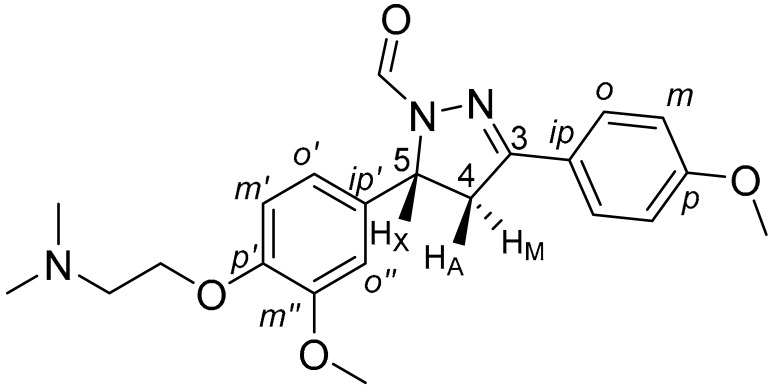
Structure and numerical assignment of compound **5d**.

The compounds **5a**–**g** were obtained as racemic mixtures and were fully characterized by FT-IR, NMR and MS measurements (see Experimental Section). The spectroscopic characterization of compound **5d** (Figure 1) was taken as an example of the compounds **5a**–**g**. The FT-IR spectrum showed the expected absorption bands at 1654 (C=O), 1595 and 1564 (C=N and C=C), 1230 and 1122 (C–O–C) cm^−1^. The ^1^H-NMR spectrum shows that the diastereotopic protons H_A_, H_M_ and H_X_ corresponding to the pyrazolinic moiety form an AMX spin system. The H_A_ appears at *δ* = 3.16 ppm as a double-doublet with coupling constants of ^2^*J* = 17.7 and ^3^*J* = 4.9 Hz, while H_M_ and H_X_ appear each one as a double-doublet at *δ* = 3.76 ppm and *δ* = 5.45 ppm with coupling constants of ^2^*J* = 17.7, ^3^*J* = 11.7 Hz and ^3^*J* = 11.7, ^3^*J* = 4.9 Hz, respectively. Similarly, the CHO signal appears as a singlet at *δ* = 8.93 ppm. The main signals in the ^13^C-NMR for the compound **5d** correspond to C-4′ at *δ* = 42.9 ppm, C-3′ at *δ* = 155.8 ppm and the CHO at *δ* = 160.1 ppm. Finally, the mass spectra of compounds **5a**–**g** showed well-defined molecular ions in all the cases.

### 2.2. Antifungal Activity

The antifungal properties of compounds **4a**–**g** and **5a**–**g** were investigated against a panel of clinically important fungal species including yeasts, *Aspergillus* spp. and dermatophytes. The minimum inhibitory concentrations (MICs) of all compounds were determined with the microbroth dilution methods M27-A3 and M38-A2 of the Clinical and Laboratory Standards Institute (CLSI) [32,33], against a panel of eight fungal species comprising two yeasts (*Candida albicans* and *Cryptococcus neoformans*), three *Aspergillus* spp. (*A. niger*, *A. fumigatus*, and *A. flavus*) and three dermatophytes (*Trichophyton rubrum*, *T. mentagrophytes* and *Microsporum gypseum*). Compounds with MICs > 250 μg/mL were considered inactive; between 250 and 125 μg/mL, with low activity and in the range 100–31.25 μg/mL, moderately active. MICs below 31.25 μg/mL were considered as indicative of high activity.

From the results of Table 1 several Structure-Activity Relationship trends can be envisaged: (i) in general, the chalcone derivatives **4** showed better activities than the pyrazolines **5** against all tested fungi. This is evidenced by the fact that only three compounds (**5a**–**c**) out of seven compounds **5** displayed antifungal activity against at least in one fungal spp., while six of seven compounds **4** compounds **4** (**4a**–**d**, **4f**, **4g**), showed antifungal properties with MICs ≤ 250 µg/mL; (ii) regarding the sensitivity of the different fungal species towards the tested compounds, it is observed that (a) none of the compound **4a**–**g** and **5a**–**g** showed activity against the three *Aspergillus* spp.; (b) only four compounds (**4a**–**c**, **5c**), out of the fourteen compounds **4** and **5**, showed activity (low, MICs 125–250 µg/mL) against *C. albicans*; (c) eight compounds (**4b**–**d**; **4f**, **4g**; **5a**–**c**) showed low (MICs 125–250 µg/mL) or moderate (**4a**, MIC = 62.5 µg/mL) activity against *Cryptococcus neoformans*; (d) compounds **4a**–**d** and **4g** showed moderate to high activities against the three dermatophytes (MICs 31.25–62.5 µg/mL), being **4a** followed by **4c** the ones that displayed the highest activity. Interestingly, the most active structures **4a**, **4c** possess fungicidal rather than fungistatic activities, with MFC values between 31.25 and 62.5 μg/mL. Compound **4a** showed the lowest MFC values against the three dermatophytes (31.25 µg/mL). In previous works Muškinja [10], Burmudžija [20], Patel [34] synthetized a series of chalcones and pyrazoline derivatives containing the vanillin moiety and tested them against several fungi including *C. albicans* and *A. niger* all of them showing negligible activity (MICs ≥ 625 µg/mL). Although the chalcone or pyrazoline derivatives reported by them differ from the series presented here in the substitution of the vanillin OH or in the ring A, the previous and presently reported compounds share the structural characteristics that they are both chalcones or pyrazolines and both possess the vanillin moiety as the B-ring, so our results add new important data to the activity of chalcone and pyrazoline vanillin derivatives possessing a vanillin moiety as ring B, corroborating that they are not good inhibitors of *C. albicans*, or *Aspergillus* spp., but adding an important finding that is their promising activity against dermatophytes and *C. neoformans*.

### 2.3. Second Order Studies of Compounds ***4*** against C. neoformans

It is worth noting that six of the seven compounds **4** showed some degree of antifungal activity against *C. neoformans*, which is the most frequent cause of HIV-related fatal opportunistic meningoencephalitis worldwide [35]. Although the incidence of this disease has tended to decline in countries with highly active anti-retroviral therapy programs, the outcome of infections is influenced by a variety of factors, including the antifungal resistance. This scenario has motivated a great interest on new anti-cryptococcal chemical structures as alternatives to the antifungal drugs currently in clinical use [36,37]. Among compounds **4**, **4a** showed the highest activity against *C. neoformans*, followed by **4b**–**d**, **4f** and **4g** that showed moderate to low activity against this yeast.

**Figure 2:**
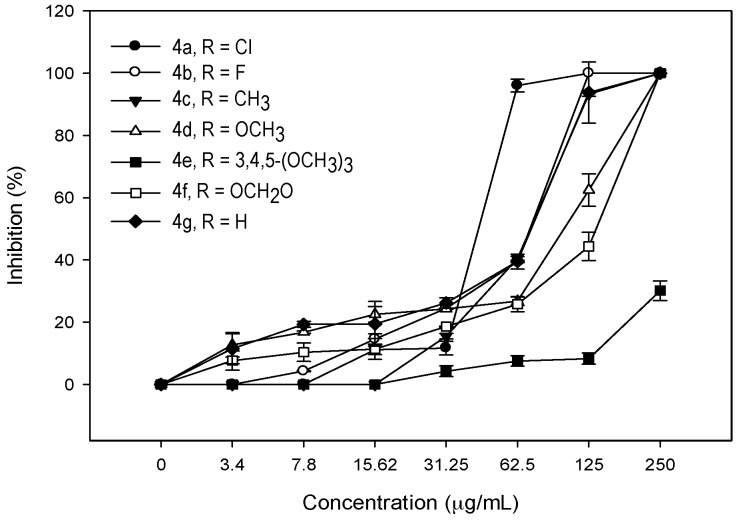
Dose-response curves of compounds **4a**–**g** against *C. neoformans* ATCC 32264.

In order to determine the influence of the A-ring substituents on the antifungal behavior of compounds **4**, the percentages of inhibition of *C. neoformans* of each compounds **4a**–**g** at concentrations from 250 to 3.9 µg/mL obtained by two-fold dilutions were determined. Results are recorded in Table 2 and represented in Figure 2, where the differences in the activity of the seven compounds **4** against *C. neoformans* can be clearly observed.

In Figure 2, the influence of the substituents of compounds **4a**–**g** on the activity against *C. neoformans* ATCC 32264 at 100 µg/mL can be clearly observed. Compounds **4a** and **4b** with electron withdrawing substituents are the most active compounds followed by **4c** and **4g** that possess a CH_3_ and a H respectively in the 4-position. Lower activities are displayed by **4d** and **4f** with electron-donating substituents such as OCH_3_ and OCH_2_O, while negligible activity was shown by the trimethoxy-substituted derivative **4e**. Regarding the difference in activity between **4a** and **4b**, **4a** showed higher activity than **4b** since it reaches almost full inhibition (96.0% ± 2.0) at 62.5 µg/mL while **4b** showed much lower inhibition percentage (39.4% ± 2.4) at the same concentration (Table 1).

In order to gain a deeper insight into the inhibitory capacity of the most anti-cryptococcal compounds **4a**–**c** and **4g** against *C. neoformans*, the four compounds were tested in a new panel of five clinical *C. neoformans* strains isolated from AIDs patients suffering from mycoses. The MICs were determined against this new panel by using three endpoints: MIC_100_, MIC_80_ and MIC_50_ (defined as the minimum concentration of compounds that inhibit 100%, 80% and 50% of growth respectively) since the application of less stringent endpoints such as MIC_80_ and MIC_50_ have shown to consistently represent the in vitro activity of the tested compounds and many times provides a better correlation with other measurements of antifungal activity [38].

Results (Table 3) showed that **4a** again displayed the best anti-cryptococcal activity against the clinical isolates, even though not highly different from the other three compounds **4b**, **4c** and **4g**. As a result of the testing of the whole series of chalcone vanillin derivatives **4** and pyrazoline vanillin derivatives **5** as antifungal agents, we could demonstrate that chalcone derivatives **4** displayed better activities than pyrazoline derivatives **5** and thus, compounds **4** could be considered as good starting points for the designing of new series of compounds with improved antifungal properties, useful for the development of new antifungal agents mainly against dermatophytes and *C. neoformans*.

### 2.4. DFT Calculations

#### 2.4.1. Chalcones vs. Pyrazolines: A Chemical Reactivity Analysis

**Figure 3:**
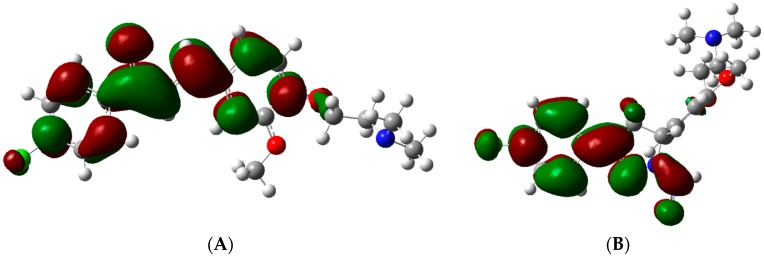
*LUMO* orbitals for compounds **4a** (**A**) and **5a** (**B**).

Despite the high structural similarity between the chalcone and pyrazoline moieties, they presented different antifungal activity profiles against several fungal species. In order to explain these biological differences, we proceeded to study electronic properties of both sets of chalcones and pyrazolines compounds through the computation of global reactivity indexes as the energy for the highest occupied molecular orbital (*E_HOMO_*), the energy for the lowest unoccupied molecular orbital (*E_LUMO_*), the chemical hardness (*η*) and softness (*S*), the electronegativity (*χ*), and the electrophilicity index (*ω*). A summary of these quantum mechanical descriptors computed for chalcone and pyrazoline compounds is presented in Table 4. The results showed significantly lower values of *E_LUMO_* for chalcones than for pyrazolines (Figure 3), being an indicative for higher reactivity for the former than the latter. Based on Koopmans’ theorem [39], the *E_LUMO_* of a molecule is directly related with its electron affinity, thus the lower the *E_LUMO_* the greater the capability to receive one electron from the environment and hence, the greater the reactivity. As a consequence, the increased antifungal activity of chalcones in comparison to pyrazolines can be attributed to the higher facility to receive electrons, particularly in their *α*,*β*-unsaturated ketone units (Figure 3), for example through a nucleophilic attack by a molecular target or disturbing the fungal redox equilibrium. The greater tendency to attract electrons by chalcones than in pyrazolines is also evidenced by the higher calculated values of *χ* for the former than in the latter. Furthermore, these arguments are supported by the analysis of additional reactivity indexes as *η*, *S* and *ω*. The maximum hardness principle indicates that when hardness increases the reactivity should decrease [40]. Due to the inverse relationship of the hardness and softness, the most reactive compound is softer. Thus, pyrazolines exhibit greater *η* values (bigger *HOMO*-*LUMO* gaps) and lower *S* values than chalcones and, subsequently a lower reactivity. Another useful quantum mechanical descriptor for explaining biological differences between chalcones and pyrazolines is the electrophilicity index (*ω*), which assess the electrophilic nature of a molecule in a relative scale. Here, chalcones showed a greater electrophilic character (higher *ω* values) than pyrazolines.

**Figure 4:**
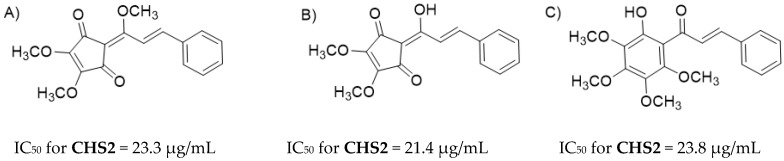
Structures of methyllinderone (**A**); linderone (**B**) and kanakugiol (**C**).

Enzymes for the construction of the fungal cell wall have been identified as potential molecular targets for chalcones, mainly the (1,3)β-d-glucan synthase and chitin synthase [41,42]. In fact, the chalcones methyllinderone, linderone and kanakugiol (Figure 4), extracted from the stem bark of *Lindera erythrocarpa* Makino, have shown potent inhibitory activity against chitin synthase 2 (CHS2) from *Saccharomyces cerevisiae* [43]. Also, they presented an antifungal activity against *C. neoformans* comparable to the antifungal activity of the synthesized chalcones here. Thus, quantum reactivity descriptors for these secondary metabolites were also computed in order to correlate their electronic structures with their biological activities (Table 4). Clearly, these compounds showed a great reactive/electrophilic profile similar to the synthetic chalcones. In particular, linderone, the most active inhibitor against CHS2, showed the lowest values of *E_LUMO_* and *η* and the highest values of *χ*, *S* and *ω* among all studied compounds. The electrophilic character of these natural products can explain their enzymatic inhibitory activity, considering that the catalytic machinery of CHS2 consists mainly of a conserved EDR motif rich in nucleophilic groups [44,45], which can attack the highly electrophilic center of chalcones. Finally, from DFT calculations it is possible to suggest a similar antifungal action mechanism (inhibition of CHS2) for synthetic chalcones and those obtained from natural sources studied here.

#### 2.4.2. Reactivity Analysis on Chalcone Series

Global reactive indexes were also applied for a quantitative analysis to evaluate the influence of the structure electronic on the antifungal activity for the chalcone series exclusively. To this aim, the computed global quantum descriptors were correlated with the negative logarithm of the MIC (pMIC) of all tested chalcones against five fungal species consisting of *C. albicans* (pMIC-Ca), *C. neoformans* (pMIC-Cn), *M. gypseum* (pMIC-Mg), *T. rubrum* (pMIC-Tr) and *T. mentagrophytes* (pMIC-Tm). A value of MIC = 250 μg/mL was assigned to inactive chalcones (MIC values > 250 μg/mL) against these five fungal species for making the correlation analysis. Because all synthetized chalcone series were inactive against the tested *Aspergillus* spp. (*A. niger*, *A. fumigatus* and *A. flavus*), they were excluded from the correlation analysis. A summary of the correlation matrix for the quantum descriptors and the antifungal activities of the chalcone series is presented in Table 5.

**Figure 5:**
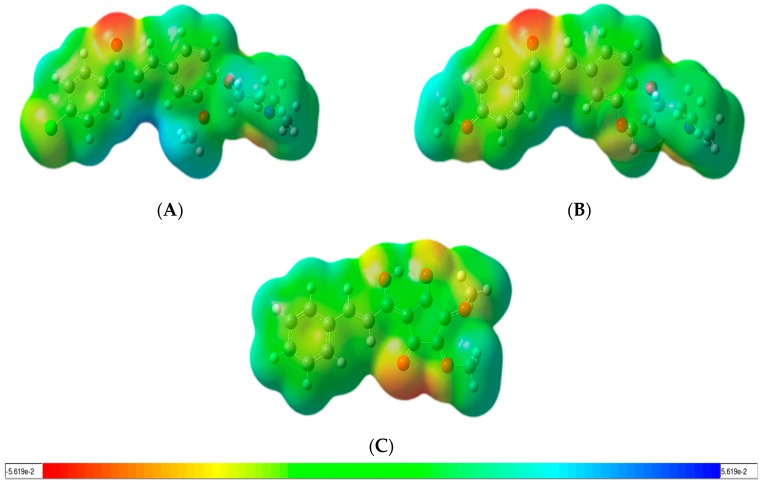
Molecular electrostatic potential maps for compounds **4a** (**A**); **4d** (**B**) and linderone (**C**).

The results showed a good correlation among the reactivity parameters *χ*, *ω*, and antifungal activities as pMIC-*C.n*., pMIC-*M.g*. and pMIC-*T.r*. Also, the *E_LUMO_* of chalcones presented an inverse correlation with their antifungal activity against yeast species (*C. albicans* and *C. neoformans*). Thus, an increase of the electrophilic character of the chalcone produces an increase on the antifungal activity. In particular, electron-withdrawing substituted chalcones (e.g., with chloride as **4a**) on the ring A were more active against fungal species than electron-donating substituted chalcones (e.g., with a methoxy group as in **4d**) in the same position because those electron-withdrawing groups increase the global electrophilic character by subtracting electron density of the electrophilic center of the chalcones through a resonance effect. This explains the antifungal activities experimentally found in the chalcones series (as **4a** > **4b**) since their computed reactivity parameters as *E_LUMO_*, *ω*, *S* in Table 4 follow the same tendency. The influence of the substituent in the electronic distribution can be visualized through the molecular electrostatic potential maps (MEP), which were computed for chalcones **4a**, **4d** and linderone (Figure 5). Along this line, a slightly lower electron density around the *α*,*β*-unsaturated ketone region is more observed in **4a** than in **4d** (Figure 5A,B). The same behavior can be observed for the studied natural chalcones as linderone (Figure 5C).

**Figure 6:**
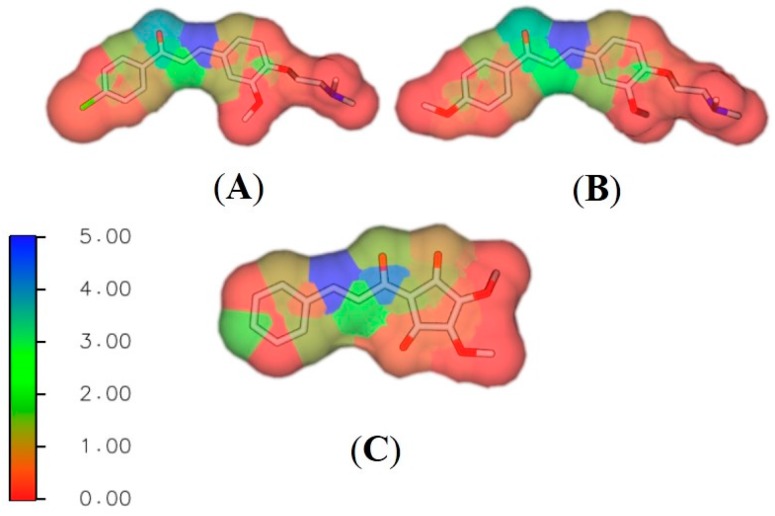
Atomic philicity index values (*ω*^+^*_k_*) projected on molecular surfaces for compounds **4a** (**A**); **4d** (**B**) and linderone (**C**).

Global reactivity indexes characterize the reactivity of a molecule as a whole and they have the same value in all regions of the molecule. In order to understand details on a reaction mechanism, apart from the global properties, local reactivity indexes are necessary for distinguish the reactive behavior of particular regions or atoms of a molecule [46]. Thus, for the *k*th atom in a molecule, the condensed Fukui functions (*f^α^_k_*, *α* = +, − or 0 for nucleophilic, electrophilic or radical attacks, respectively), the local softness (*s^α^_k_*) and philicity indexes (*ω^α^_k_*) are the most common local parameters used in chemical reactivity analysis. Here, these local reactivity parameters have been computed for the chalcone series in order to explore the most reactive sites for each molecule. Considering the electrophilic nature of the chalcone moiety, we have focused the analysis on local philicity indexes for nucleophilic attacks (*ω*^+^*_k_*), since the other local parameters might provide similar conclusions. Figure 6 shows the condensed *ω*^+^*_k_* values projected on molecular surfaces for chalcones **4a**, **4d** and linderone.

Atoms or sites in a molecule with high susceptibility to nucleophilic attacks present higher values of *ω*^+^ (blue regions in Figure 6). Clearly, for compounds 4a and 4d, the most preferred site for a nucleophilic attack is the *β* carbon at the *α*,*β*-unsaturated carbonyl group (Figure 6A,B). The *β* carbon even shows a higher reactivity than the carbon atoms *α* and carbonyl in the synthetic chalcones. In the case of linderone, the most reactive atom is still the *β* carbon at the enol group, although a second highly reactive site suitable for nucleophilic attacks appears at the enolic carbon (Figure 6C).

## 3. Experimental Section

### 3.1. General Information

Solvents and reagents were purchased from Sigma-Aldrich (St. Louis, MO, USA) or Merck Millipore (Billerica, MA, USA) and were used without purification. Thin layer chromatography (TLC) was performed on 0.2-mm pre-coated plates of silica gel 60GF254 (Merck). Melting points were measured using an SMP3 melting point device (Stuart, Staffordshire, UK). FT-IR spectra were obtained with a Thermo Scientific (Waltham, MA, USA) Nicolet 6700 equipped with ATR. The ^1^H- and ^13^C-NMR spectra were run on a DPX 400 spectrometer (Bruker, Billerica, MA, USA) operating at 400 and 100 MHz, respectively, using CDCl_3_ as solvent and TMS as internal standard. The mass spectra were obtained on a GCMS-QP2010 spectrometer (Shimadzu, Kyoto, Japan) operating at 70 eV. The elemental analyses were obtained using a Thermo Finnigan (Somerset, NJ, USA) Flash EA1112 CHN (STIUJA) elemental analyzer.

### 3.2. Synthesis

#### 3.2.1. Synthesis of the Precursor 4-(2-(Dmethylamino)ethoxy)-3-methoxybenzaldehyde (**2**)

A mixture of vanillin (**1**, 13.16 mmol, 2 g), 2-chloro-*N*,*N*-dimethylethan-1-amine hydrochloride (18.72 mmol, 2.8 g), in DMF (20 mL), was subjected to refluxing with continuous stirring for 24 h in the presence of anhydrous potassium carbonate (52 mmol, 7.2 g). The reaction progress was monitored by TLC. After completion of the reaction, the resultant suspension was cooled to room temperature, quenched with cold water and the crude was extracted with CHCl_3_. Then the organic phase was treated with anhydrous magnesium sulfate and the solvent was eliminated under reduced pressure. A dark oil was obtained, which was purified by column chromatography on silica gel employing 30:1 CHCl_3_-MeOH as eluent. Brown oil, 1.47 g, 50% yield; ^1^H-NMR (CDCl_3_) *δ* 9.87 (s, 1H, CHO), 7.46 (dd, *J* = 8.1, 1.8 Hz, 1H, H-6), 7.43 (d, *J* = 1.8 Hz, 1H, H-2), 7.01 (d, *J* = 8.1 Hz, 1H, H-5), 4.22 (t, *J* = 6.0 Hz, 2H, O-CH_2_), 3.94 (s, 3H, O-CH_3_), 2.85 (t, *J* = 6.0 Hz, 2H, N-CH_2_), 2.38 (s, 6H, 2N-CH_3_).

#### 3.2.2. General Process for the Synthesis of Compounds **4a**–**g**

A mixture of aldehyde **2** (3.1 mmol, 700 mg), the appropriate commercially available substituted acetophenone **3a**–**f** (3.7 mmol) and 20% aq. NaOH (1 mL) in MeOH (10 mL) was stirred at room temperature for 30 min. The reaction progress was monitored by TLC. After completion of reaction, the reaction mixture was quenched with water and extracted with CHCl_3_. Then the organic phase was treated with anhydrous magnesium sulfate and the solvent was eliminated under reduced pressure. A dark oil was obtained, which was purified by column chromatography on silica gel employing 15:1 of CHCl_3_-MeOH as eluent. All the compounds were obtained as red oils.

*(E)-1-(4-Chlorophenyl)-3-(4-(2-(dimethylamino)ethoxy)-3-methoxyphenyl)prop-2-en-1-one* (**4a**)*.* 1.04 g, 92% yield. FTIR (ATR) *υ* (cm^−1^): 2943 (C-H), 1658 (C=O), 1588 (C=C), 1209 (C-N), 1257 y 1027 (C-O-C). ^1^H-NMR (CDCl_3_) *δ* 7.95 (d, *J* = 8.6 Hz, 2H, H-*o*), 7.75 (d, *J* = 15.6 Hz, 1H, H-*β*), 7.46 (d, *J* = 8.6 Hz, 2H, H-*m*), 7.32 (d, *J* = 15.6 Hz, 1H, H-*α*), 7.21 (dd, *J* = 8.3, 1.9 Hz, 1H, H-*o*′), 7.14 (d, *J* = 1.9 Hz, 1H, H-*o*″), 6.90 (d, *J* = 8.3 Hz, 1H, H-*m*′), 4.15 (t, *J* = 6.0 Hz, 2H, O-CH_2_), 3.92 (s, 3H, O-CH_3_), 2.80 (t, *J* = 6.0 Hz, 2H, N-CH_2_), 2.35 (s, 6H, 2 × N-CH_3_). ^13^C-NMR (CDCl_3_) *δ* 189.7 (C=O), 151.4 (C-*ip*), 150.1 (C-*p*′), 145.9 (C-*m*″), 139.4 (C-*β*), 137.2 (C-*p*), 130.2 (C-*o*), 130.0 (C-*ip*′), 129.3 (C-*o*′), 123.6 (C-*α*), 119.9 (C-*m*), 113.1 (C-*m*′), 111.1 (C-*o*″), 67.4 (O-CH_2_), 58.4 (N-CH_2_), 56.5 (O-CH_3_), 46.3 (N-CH_3_). MS (70 eV) *m*/*z* (%): 359/361 [M^+^] (13/4), 139 (58), 111 (51), 72 (100), 58 (99). Anal. Calcd. For C_20_H_22_ClNO_3_: C, 66.76; H, 6.16; N, 3.89. Found: C, 66.77; H, 6.14; N, 3.88.

*(E)-3-(4-(2-(Dimethylamino)ethoxy)-3-methoxyphenyl)-1-(4-fluorophenyl)prop-2-en-1-one* (**4b**)*.* 0.88 g, 82% yield. FTIR (ATR) *υ* (cm^−1^): 2943 (C-H), 1659 (C=O), 1596 (C=C), 1233 (C-N), 1257 y 1025 (C-O-C). ^1^H-NMR (CDCl_3_) *δ* 8.04 (dd, *J* = 8.8, 5.4 Hz, 2H, H-*o*), 7.75 (d, *J* = 15.6 Hz, 1H, H-*β*), 7.35 (d, *J* = 15.6 Hz, 1H, H-*α*), 7.21 (dd, *J* = 8.3, 1.9 Hz, 1H, H-*o*′), 7.19–7.13 (m, 3H, H*m*, H-*o*″), 6.91 (d, *J* = 8.3 Hz, 1H, H-*m*′), 4.16 (t, *J* = 6.0 Hz, 2H, O-CH_2_), 3.92 (s, 3H, O-CH_3_), 2.80 (t, *J* = 6.0 Hz, 2H, N-CH_2_), 2.35 (s, 6H, 2 × N-CH_3_). ^13^C-NMR (CDCl_3_) *δ* 189.0 (C=O), 165.7 (d, *J_C-F_* = 251.0 Hz, C-*p*), 151.0 (d, *J_C–F_* = 9.1 Hz, C-*ip*), 149.8 (C-*p*′), 149.5 (C-*m*″), 134.9 (C-*β*), 131.2 (d, *J*_C−F_ = 21.3 Hz, C-*m*), 128.1 (C-*ip*′), 123.2 (C-*o*′), 119.7 (C-*α*), 115.8 (d, *J*_C−F_ = 9.2 Hz, C-*o*), 112.8 (C-*m*′), 110.8 (C-*o*″), 67.1 (O-CH_2_), 58.1 (N-CH_2_), 56.1 (O-CH_3_), 46.0 (N-CH_3_). MS (70 eV) *m*/*z* (%): 343 [M^+^] (15), 72 (33), 58 (100). Anal. Calcd. For C_20_H_22_FNO_3_: C, 69.95; H, 6.46; N, 4.08. Found: C, 69.93; H, 6.47; N, 4.07.

*(E)-3-(4-(2-(Dimethylamino)ethoxy)-3-methoxyphenyl)-1-(p-tolyl)prop-2-en-1-one* (**4c**). 0.90 g, 85% yield. FTIR (ATR) *υ* (cm^−1^): 2945 (C-H), 1660 (C=O), 1580 (C=C), 1250 y 1045 (C-O-C). ^1^H-NMR (CDCl_3_) *δ* 7.92 (d, *J* = 8.2 Hz, 2H, H-*o*), 7.74 (d, *J* = 15.6 Hz, 1H, H-*β*), 7.38 (d, *J* = 15.6 Hz, 1H, H-*α*), 7.29 (d, *J* = 8.0 Hz, 2H, H-*m*), 7.21 (dd, *J* = 8.3, 1.9 Hz, 1H, H-*o*′), 7.15 (d, *J* = 1.9 Hz, 1H, H-*o*″), 6.90 (d, *J* = 8.3 Hz, 1H, H-*m*′), 4.16 (t, *J* = 6.1 Hz, 2H, O-CH_2_), 3.92 (s, 3H, O-CH_3_), 2.80 (t, *J* = 6.0 Hz, 2H, N-CH_2_), 2.43 (s, 3H, CH_3_), 2.35 (s, 6H, 2 × N-CH_3_). ^13^C-NMR (CDCl_3_) *δ* 190.2 (C=O), 150.8 (C-*p*), 149.7 (C-*p*′), 144.7 (C-*m*″), 143.5 (C-*β*), 136.0 (C-*ip*), 129.4 (C-*o*), 128.7 (C-*ip*′), 128.3 (C-*o*′), 123.0 (C-*α*), 120.3 (C-*m*), 112.8 (C-*m*′), 110.8 (C-*o*″), 67.1 (O-CH_2_), 58.1 (N-CH_2_), 56.1 (O-CH_3_), 46.0 (N-CH_3_), 21.8 (CH_3_). MS (70 eV) *m*/*z* (%): 339 [M^+^] (15), 72 (45), 58 (100). Anal. Calcd. For C_21_H_25_NO_3_: C, 74.31; H, 7.42; N, 4.13. Found: C, 74.30; H, 7.45; N, 4.12.

*(E)-3-(4-(2-(Dimethylamino)ethoxy)-3-methoxyphenyl)-1-(4-methoxyphenyl)prop-2-en-1-one* (**4d**)*.* 1.04 g, 93% yield. FTIR (ATR) *υ* (cm^−1^): 2940 (C-H), 1654 (C=O), 1596 (C=C), 1253 y 1020 (C-O-C). ^1^H-NMR (CDCl_3_) *δ* 8.03 (d, *J* = 8.9 Hz, 2H, H-*o*), 7.74 (d, *J* = 15.6 Hz, 1H, H-*β*), 7.39 (d, *J* = 15.6 Hz, 1H, H-*α*), 7.20 (dd, *J* = 8.3, 1.9 Hz, 1H, H-*o*′), 7.15 (d, *J* = 1.9 Hz, 1H, H-*o*″), 6.98 (d, *J* = 8.8 Hz, 2H, H-*m*), 6.90 (d, *J* = 8.3 Hz, 1H, H-*m*′), 4.15 (t, *J* = 6.0 Hz, 2H, O-CH_2_), 3.92 (s, 3H, O-CH_3_), 3.88 (s, 3H, O-CH_3_), 2.80 (t, *J* = 6.0 Hz, 2H, N-CH_2_), 2.34 (s, 6H, 2 × N-CH_3_). ^13^C-NMR (CDCl_3_) *δ* 188.9 (C=O), 163.4 (C-*ip*), 150.7 (C-*p*′), 149.7 (C-*m*″), 144.2 (C-*β*), 131.5 (C-*p*), 130.9 (C-*o*), 128.4 (C-*ip*′), 122.9 (C-*o*′), 120.1 (C-*α*), 113.9 (C-*m*), 112.8 (C-*m*′), 110.8 (C-*o*″), 67.1 (O-CH_2_), 58.1 (N-CH_2_), 56.1 (O-CH_3_), 55.6 (O-CH_3_), 46.0 (N-CH_3_). MS (70 eV) *m*/*z* (%): 355 [M^+^] (20), 135 (10), 72 (56), 58 (100). Anal. Calcd. For C_21_H_25_NO_4_: C, 70.96; H, 7.09; N, 3.94. Found: C, 70.97; H, 7.07; N, 3.96.

*(E)-3-(4-(2-(Dimethylamino)ethoxy)-3-methoxyphenyl)-1-(3,4,5-trimethoxyphenyl)prop-2-en-1-one* (**4e**)*.* 1.10 g, 85% yield. FTIR (ATR) *υ* (cm^−1^): 2943 (C-H), 1648 (C=O), 1580 (C=C), 1216 y 1024 (C-O-C). ^1^H-NMR (CDCl_3_) *δ* 7.75 (d, *J* = 15.6 Hz, 1H, H-*β*), 7.32 (d, *J* = 15.6 Hz, 1H, H-*α*), 7.26 (d, *J* = 1.2 Hz, 2H, H-*m*), 7.23 (dd, *J* = 8.3, 1.9 Hz, 1H, H-*o*′), 7.14 (d, *J* = 1.9 Hz, 1H, H-*o*″), 6.92 (d, *J* = 8.3 Hz, 1H, H-*m*′), 4.16 (t, *J* = 6.0 Hz, 2H, O-CH_2_), 3.97–3.89 (m, 12H, 4O-CH_3_), 2.80 (t, *J* = 6.0 Hz, 2H, N-CH_2_), 2.35 (s, 6H, 2 × N-CH_3_). ^13^C-NMR (CDCl_3_) *δ* 189.6 (C=O), 153.3 (C-*p*), 150.9 (C-*p*′), 149.8 (C-*m*″), 145.1 (C-*β*), 142.6 (C-*ip*), 133.9 (C-*o*), 128.2 (C-*ip*′), 122.9 (C-*o*′), 120.1 (C-*α*), 112.9 (C-*m*), 111.2 (C-*m*′), 106.3 (C-*o*″), 67.1 (O-CH_2_), 61.1 (O-CH_3_), 58.1 (N-CH_2_), 56.6 (O-CH_3_), 56.2 (OCH_3_), 46.0 (N-CH_3_). MS (70 eV) *m*/*z* (%): 415 [M^+^] (45), 72 (42), 58 (100). Anal. Calcd. For C_23_H_29_NO_6_: C, 66.49; H, 7.04; N, 3.37. Found: C, 66.50; H, 7.03, N, 3.37.

*(E)-1-(Benzo[d][1,3]dioxol-5-yl)-3-(4-(2-(dimethylamino)ethoxy)-3-methoxyphenyl)prop-2-en-1-one* (**4f**). 1.00 g, 87% yield. FTIR (ATR) *υ* (cm^−1^): 2942 (C-H), 1654 (C=O), 1564 (C=C), 1245 y 1021 (C-O-C). ^1^H-NMR (CDCl_3_) *δ* 7.73 (d, *J* = 15.5 Hz, 1H, H-*β*), 7.64 (dd, *J* = 8.2, 1.7 Hz, 1H, H-*o*′′′), 7.52 (d, *J* = 1.7 Hz, 1H, H-*o*), 7.34 (d, *J* = 15.5 Hz, 1H, H-*α*), 7.19 (dd, *J* = 8.3, 1.9 Hz, 1H, H-*o*′), 7.14 (d, *J* = 1.9 Hz, 1H, H-*o*″), 6.90–6.88 (m, 2H, H-*m* y H-*m*′), 6.05 (s, 2H, -OCH_2_O-), 4.18 (t, *J* = 6.1 Hz 2H, O-CH_2_), 3.92 (s, 3H, O-CH_3_), 2.82 (t, *J* = 6.1 Hz, 2H, N-CH_2_), 2.37 (s, 6H, 2 × N-CH_3_). ^13^C-NMR (CDCl_3_) *δ* 190.7 (C=O), 152.8 (C-*p*), 151.1 (C-*p’*), 149.9 (C-*m*″), 148.2 (C-*β*), 144.3 (C-*ip*), 132.6 (C-*o*), 129.7 (C-*ip*′), 123.2 (C-*o*′), 122.4 (C-*α*), 114.3 (C-*m*), 111.6 (C-*m*′), 110.5 (C-*o*′′′), 108.6 (C-*o*″), 102.1 (O-CH2-O), 68.3 (O-CH_2_), 58.4 (N-CH_2_), 56.8 (O-CH_3_), 45.6 (N-CH_3_). MS (70 eV) *m*/*z* (%): 369 [M^+^] (40), 149 (62), 72 (100) 58 (99). Anal. Calcd. For C_21_H_23_NO_5_: C, 68.28; H, 6.28; N, 3.79. Found: C, 68.26; H, 6.29; N, 3.78.

*(E)-3-(4-(2-(Dimethylamino)ethoxy)-3-methoxyphenyl)-1-phenylprop-2-en-1-one* (**4g**)*.* 0.76 g, 78% yield. FTIR (ATR) *υ* (cm^−1^): 2951 (C-H), 1658 (C=O), 1592 (C=C), 1255 y 1017 (C-O-C). ^1^H-NMR (CDCl_3_) *δ* 8.01 (d, *J* = 7.0 Hz, 2H, H-*o*), 7.75 (d, *J* = 15.6 Hz, 1H, H-*β*), 7.58 (t, *J* = 8.0 Hz, 1H, Ar-H), 7.50 (dd, *J* = 8.0, 7.0 Hz 2H, H-*m*), 7.38 (d, *J* = 15.6 Hz, 1H, H-*α*), 7.21 (dd, *J* = 8.3, 1.9 Hz, 1H, H-*o*′), 7.15 (d, *J* = 1.9 Hz, 1H, H-*o*″), 6.91 (d, *J* = 8.3 Hz, 1H, H-*m*′), 4.16 (t, *J* = 6.1 Hz, 2H, O-CH_2_), 3.92 (s, 3H, O-CH_3_), 2.80 (t, *J* = 6.0 Hz, 2H, N-CH_2_), 2.35 (s, 6H, 2 × N-CH_3_). ^13^C-NMR (CDCl_3_) *δ* 190.8 (C=O), 150.9 (C-*p*), 149.8 (C-*p*′), 145.2 (C-*m*″), 138.7 (C-*β*), 132.7 (C-*ip*), 128.7 (C-*o*), 128.6 (C-*ip*′), 128.2 (C-*o*′), 123.2 (C-*α*), 120.3 (C-*m*), 112.9 (C-*m*′), 110.8 (C-*o*″), 67.2 (O-CH_2_), 58.1 (N-CH_2_), 56.1 (O-CH_3_), 46.1 (N-CH_3_). MS (70 eV) *m*/*z* (%): 325 [M^+^] (8), 72 (100), 58 (99). Anal. Calcd. For C_20_H_23_NO_3_: C, 73.82; H, 7.12; N, 4.30. Found: C, 73.83, H, 7.12, N, 4.31.

#### 3.2.3. General Process for the Synthesis of Compounds **5a**–**g**

A mixture of chalcone **4a**–**g** (0.23 mmol), hydrazine hydrate (0.5 mmol) and formic acid (2 mL) was subjected to reflux for 2–4 h with continuous stirring. The reaction progress was monitored by TLC. After completion of reaction, ethanol was added and the precipitate formed was filtered and discarded. Then, the filtered was extracted using CHCl_3_, the organic phase was treated with anhydrous magnesium sulfate and the solvent was eliminated under reduced pressure. The oils obtained were washed with ethyl ether to form the compounds **5a**–**g** as beige solids.

*3-(4-Chlorophenyl)-5-(4-(2-(dimethylamino)ethoxy)-3-methoxyphenyl)-4,5-dihydro-1H-pyrazole-1-carb-aldehyde* (**5a**)*.* 67 mg, 73% yield; m.p.: 106–107 °C. FTIR (ATR) *υ* (cm^−1^): 2922 y 2725 (C-H), 1655 (C=O), 1595 y 1512 (C=C y C=N). ^1^H-NMR (CDCl_3_) *δ* 8.93 (s, 1H, CHO), 7.66 (d, *J* = 8.5 Hz, 2H, H-*o*), 7.40 (d, *J* = 8.5 Hz, 2H, H-*m*), 6.89 (d, *J* = 8.8 Hz, 1H, H-*m*′), 6.81–6.79 (m, 2H, H-*o*′ y H-*o*″), 5.48 (dd, *J* = 11.7, 5.0 Hz, 1H, H_X_), 4.47 (t, *J* = 6.0 Hz 2H, O-CH_2_), 3.81 (s, 3H, O-CH_3_), 3.74 (dd, *J* = 11.7, 5.0 Hz, 1H, H_M_), 3.47 (t, *J* = 6.0 Hz 2H, N-CH_2_), 3.16 (dd, *J* = 17.8, 5.0 Hz, 1H, H_A_), 2.93 (s, 6H, 2 × N-CH_3_). ^13^C-NMR (CDCl_3_) *δ* 160.2 (CHO), 154.8 (C-*p*), 150.3 (C-3), 146.5 (C-*m*″), 136.8 (C-*p*′), 135.4 (C-*ip*′), 129.3 (C-*o*), 129.2 (C-*ip*), 127.9 (C-*o*′), 117.9 (C-*m*′), 115.7 (C-*m*), 109.6 (C-*o*″), 64.8 (O-CH_2_), 59.0 (C-5), 56.5 (N-CH_2_), 55.9 (O-CH_3_), 43.7 (N-CH_3_), 42.6 (CH_2_). MS *m*/*z* 401/403 [M^+^] (100/38), 72 (14), 58 (44). Anal. Calcd. For C_21_H_24_ClN_3_O_3_: C, 62.79; H, 6.02; N, 10.46. Found: C, 62.80; H, 6.01; N, 10.46.

*5-(4-(2-(Dimethylamino)ethoxy)-3-methoxyphenyl)-3-(4-fluorophenyl)-4,5-dihydro-1H-pyrazole-1-carb-aldehyde* (**5b**)*.* 53 mg, 60% yield; m.p.: 95–96 °C. FTIR (ATR) *υ* (cm^−1^): 2922 y 2854 (C-H), 1672 (C=O), 1604 y 1514 (C=C y C=N). ^1^H-NMR (CDCl_3_) *δ* 8.95 (s, 1H, CHO), 7.77–7.67 (m, 2H, H-*o*), 7.13 (t, *J* = 8,5, 2H, H-*m*), 6.84 (d, *J* = 8.2 Hz, 1H, H-*m*′), 6.77 (d, *J* = 11 Hz, 2H, H-*o*′ y H-*o*″), 5.49 (dd, *J* = 11.7, 4.8 Hz, 1H, H_X_), 4.08 (t, *J* = 6,0 Hz, 2H, O-CH_2_), 3.83 (s, 3H, O-CH_3_), 3.74 (dd, *J* = 17.2, 5.4 Hz, 1H, H_M_), 3.19 (dd, *J* = 17.2, 4.8 Hz, 1H, H_A_), 2.76 (t, *J* = 6.0 Hz, 2H, N-CH_2_), 2.34 (s, 6H, 2 × N-CH_3_). ^13^C-NMR (CDCl_3_) *δ* 160.2 (CHO), 154.8 (C-3), 150.5 (C-*m*″), 149.2 (d, *J*_C−F_ = 231.0 Hz, C-*p*), 133.7 (C-*p*′), 132.6 (C-*ip*′), 131.0 (d, *J*_C−F_ = 4.2 Hz, C-*ip*), 128.9 (d, *J*_C−F_ = 8.4 Hz, C-*o*), 128.9 (C-*o*′), 117.9 (C-*m*′), 116.2 (d, *J*_C−F_ = 22.1 Hz, C-*m*), 113.8 (C-*o*″), 68.3 (O-CH_2_), 63.7 (C-5), 59.1 (O-CH_3_), 58.1 (N-CH_2_), 56.1 (N-CH_3_), 45.92 (CH_2_). MS *m*/*z* 385 [M^+^] (15), 72(50), 58(100). Anal. Calcd. For C_21_H_24_FN_3_O_3_: C, 65.44; H, 6.28; N, 10.90. Found: C, 65.42; H, 6.02; N, 10.91.

*5-(4-(2-(Dimethylamino)ethoxy)-3-methoxyphenyl)-3-(p-tolyl)-4,5-dihydro-1H-pyrazole-1-carbaldehyde* (**5c**)*.* 59 mg, 67% yield; m.p.: 101–102 °C. FTIR (ATR) *υ* (cm^−1^): 2951 y 2783 (C-H), 1668 (C=O), 1595 y 1519 (C=C y C=N). ^1^H-NMR (CDCl_3_) *δ* 8.95 (s, 1H, CHO), 7.63 (d, *J* = 8.1 Hz, 2H, H-*o*), 7.24 (d, *J* = 8.1 Hz, 2H, H-*m*), 6.84 (d, *J* = 8.2 Hz, 1H, H-*m*′), 6.81–6.74 (m, 2H, H-*o*′ y H-*o*″), 5.47 (dd, *J* = 11.7, 4.7 Hz, 1H, H_X_), 4.10 (t, *J* = 5.9 Hz, 2H, O-CH_2_), 3.81 (s, 3H, O-CH_3_), 3.76 (dd, *J* = 11.7, 4.7 Hz 1H, H_M_), 3.20 (dd, *J* = 17.7, 4.7 Hz, 1H, H_A_), 2.81 (t, *J* = 5.9 Hz, 2H, N-CH_2_), 2.40 (s, 3H, CH_3_), 2.37 (s, 6H, 2 × N-CH_3_). ^13^C-NMR (CDCl_3_) *δ* 160.2 (CHO), 154.9 (C-3), 150.1 (C-*m*″), 149.1 (C-*p*), 141.2 (C-*p*′), 134.1 (C-*ip*′), 129.7 (C-*o*), 128.3 (C-*ip*), 126.8 (C-*o*′), 118.0 (C-*m*′), 113.9 (C-*m*), 109.6 (C-*o*″), 66.9 (O-CH_2_), 58.9 (C-5), 57.9 (N-CH_2_), 56.1 (O-CH_3_), 45.7 (N-CH_3_), 42.9 (CH_2_), 21.7 (CH_3_). MS *m*/*z* 381 [M^+^] (20), 72(46), 58(100)*.* Anal. Calcd. For C_22_H_27_N_3_O_3_: C, 69.27; H, 7.13; N, 11.01. Found: C, 69.29; H, 7.12; N, 11.03.

*5-(4-(2-(Dimethylamino)ethoxy)-3-methoxyphenyl)-3-(4-methoxyphenyl)-4,5-dihydro-1H-pyrazole-1-carb-aldehyde* (**5d**)*.* 60 mg, 66% yield; m.p.: 185–186 °C. FTIR (ATR) *υ* (cm^−1^): 2926 y 2845 (C-H), 1668 (C=O), 1602 y 1516 (C=C y C=N). ^1^H-NMR (CDCl_3_) *δ* 8.93 (s, 1H, CHO), 7.67 (d, *J* = 8.8 Hz, 2H, H-*o*), 6.94 (d, *J* = 8.8 Hz, 2H, H-*m*), 6.88 (d, *J* = 8.3 Hz, 1H, H-*m*′), 6.79–6.77 (m, 2H, H-*o*′ y H-*o*″), 5.45 (dd, *J* = 11.7, 4.9 Hz, 1H, H_X_), 4.42 (t, *J* = 4.6 Hz, 2H, O-CH_2_), 3.85 (s, 3H, O-CH_3_), 3.80 (s, 3H, OCH_3_), 3.76 (dd, *J* = 17.7, 11.7 Hz, 1H, H_M_), 3.40 (t, *J* = 4.6 Hz, 2H, N-CH_2_), 3.16 (dd, *J* = 17.7, 4.9 Hz, 1H, H_A_), 2.89 (s, 6H, 2 × N-CH_3_). ^13^C-NMR (CDCl_3_) *δ* 161.8 (C-*p*), 160.1 (CHO), 155.8 (C-3), 150.3 (C-*m*″), 146.5 (C-*p*′), 135.8 (C-*ip*′), 128.5 (C-*o*), 123.5 (C-*ip*), 118.1 (C-*o*′), 115.7 (C-*m*′), 114.4 (C-*m*), 109.7 (C-*o*″), 64.9 (O-CH_2_), 58.8 (C-5), 56.6 (N-CH_2_), 55.9 (O-CH_3_), 55.6 (O-CH_3_), 43.8 (N-CH_3_), 42.9 (CH_2_). MS *m*/*z* 397 [M^+^] (2), 347(65), 48(100). Anal. Calcd. For C_22_H_27_N_3_O_4_: C, 66.48; H, 6.85; N, 10.57. Found: C, 66.46; H, 6.85; N, 10.56.

*5-(4-(2-(Dimethylamino)ethoxy)-3-methoxyphenyl)-3-(3,4,5-trimethoxyphenyl)-4,5-dihydro-1H-pyrazole-1-carbaldehyde* (**5e**). 65 mg, 62% yield; m.p.: 219–220 °C. FTIR (ATR) *υ* (cm^−1^): 2939 y 2835 (C-H), 1660 (C=O), 1597 y 1571 (C=C y C=N). ^1^H-NMR (CDCl_3_) *δ* 8.95 (s, 1H, CHO), 6.96 (s, 2H, H-*o*), 6.89 (d, *J* = 8.3 Hz, 1H, H-*m*′), 6.78 (d, *J* = 7.5 Hz, 2H, H-*o*′ y H-*o*″), 5.48 (dd, *J* = 11.7, 5.0 Hz, 1H, H_X_), 4.40 (t, *J* = 4.7 Hz, 2H, O-CH_2_), 3.91 (s, 6H, *m*-OCH_3_), 3.89 (s, 3H, O-CH_3_), 3.82 (s, 3H, O-CH_3_), 3.79–3.73 (m, 1H, H_M_), 3.35 (t, *J* = 4.7 Hz, 2H, N-CH_2_), 3.18 (dd, *J* = 17.6, 5.0 Hz, 1H, H_A_), 2.86 (s, 6H, 2 × N-CH_3_). ^13^C-NMR (CDCl_3_) *δ* 160.4 (CHO), 156.3 (C-*p*), 153.6 (C-3), 150.7 (C-*m*″), 147.3 (C-*p*′), 141.0 (C-*ip*′), 137.4 (C-*o*), 126.8 (C-*ip*), 118.6 (C-*o*′), 116.3 (C-*m*′), 110.3 (C-*m*), 104.2 (C-*o*″), 65.4 (O-CH_2_), 61.5 (C-5), 59.2 (N-CH_2_), 57.1 (*m*-OCH_3_), 56.5 (O-CH_3_), 56.2 (O-CH_3_), 44.1 (N-CH_3_), 43.2 (CH_2_). MS *m*/*z* 457 [M^+^] (40), 72(73), 58(100). Anal. Calcd. For C_24_H_31_N_3_O_6_: C, 63.00; H, 6.83; N, 9.18. Found: C, 62.98; H, 6.84; N, 9.19.

*3-(Benzo[d][1,3]dioxol-5-yl)-5-(4-(2-(dimethylamino)ethoxy)-3-methoxyphenyl)-4,5-dihydro-1H-pyrazole-1-carbaldehyde* (**5f**). 61 mg, 65% yield; m.p.: 209–210 °C. FTIR (ATR) *υ* (cm^−1^): 2922 y 2873 (C-H), 1654 (C=O), 1606 y 1504 (C=C y C=N). ^1^H-NMR (CDCl_3_) *δ* 8.91 (s, 1H, CHO), 7.34 (s, 1H, H-*o*), 7.10 (d, *J* = 8.1 Hz, 1H, H-*o*′′′), 6.88 (d, *J* = 8.6 Hz, 1H), 6.83 (d, *J* = 8.1 Hz, 1H, H-*m*), 6.77 (d, *J* = 7.1 Hz, 2H, H-*o*′ y H-*o*″), 6.02 (s, 2H, O-CH_2_-O), 5.45 (dd, *J* = 11.7, 4.9 Hz, 1H, H_X_), 4.44–4.37 (m, 2H, O-CH_2_), 3.81 (s, 3H, O-CH_3_), 3.73 (dd, *J* = 17.6, 11.7 Hz, 1H, H_M_), 3.40–3.35 (m, 2H), 3.13 (dd, *J* = 17.6, 4.9 Hz, 1H, H_A_), 2.87 (s, 6H, 2 × N-CH_3_). ^13^C-NMR (CDCl_3_) *δ* 160.1 (CHO), 155.6 (C-*p*), 150.3 (C-3), 150.1 (C-*m*″), 148.5 (C-*m*), 146.8 (C-*p*′), 135.6 (C-*ip*′), 125.2 (C-*ip*), 122.0 (C-*o*′), 118.0 (C-*o*′′′), 115.9 (C-*m’*), 109.7 (C-*o*), 108.5 (C-*m*′′′), 106.1 (C-*o*″), 101.8 (O-CH2-O), 65.0 (O-CH_2_), 58.9 (C-5), 56.6 (N-CH_2_), 55.9 (O-CH_3_), 43.9 (N-CH_3_), 43.1 (CH_2_). MS *m*/*z* 411 [M^+^] (100), 72(33), 58(79)*.* Anal. Calcd. For C_22_H_25_N_3_O_5_: C, 64.22; H, 6.12; N, 10.21. Found: C, 64.22; H, 6.14; N, 10.19.

*5-(4-(2-(Dimethylamino)ethoxy)-3-methoxyphenyl)-3-phenyl-4,5-dihydro-1H-pyrazole-1-carbaldehyde* (**5g**)*.* 57 mg, 68% yield; m.p.: 94–95 °C. FTIR (ATR) *υ* (cm^−1^): 2922 y 2876 (C-H), 1664 (C=O), 1600 y 1554 (C=C y C=N). ^1^H-NMR (CDCl_3_) *δ* 8.96 (s, 1H, CHO), 7.74 (d, *J* = 7.7 Hz, 2H, H-*o*), 7.44–7.42 (m, 3H, H-*m* y H-*p*), 6.89 (d, *J* = 8.1 Hz, 1H, H-*m*′), 6.79–6.77 (m, 2H, H-*o*′ y H-*o*″), 5.48 (dd, *J* = 11.7, 4.9 Hz, 1H, H_X_), 4.42 (t, *J* = 4.6 Hz, 2H, O-CH_2_-O), 3.85–3.75 (m, 4H, H-4 y H_M_), 3.40 (t, *J* = 4.6 Hz, 2H, N-CH_2_), 3.21 (dd, *J* = 17.7, 4.9 Hz, 1H, H_A_), 2.90 (s, 6H, 2 × N-CH_3_). ^13^C-NMR (CDCl_3_) *δ* 160.3 (CHO), 155.9 (C-*p*), 150.3 (C-3), 146.6 (C-*m*″), 136.9 (C-*p*′), 135.7 (C-*ip*′), 130.9 (C-*o*), 129.0 (C-*ip*), 126.8 (C-*o*′), 118.1 (C-*m*′), 115.8 (C-*m*), 109.7 (C-*o*″), 64.9 (O-CH_2_), 58.9 (C-5), 56.6 (N-CH_2_), 55.9 (O-CH_3_), 43.8 (N-CH_3_), 42.8 (CH_2_). MS *m*/*z* 367 [M^+^] (15), 72(100), 58(90). Anal. Calcd. For C_21_H_25_N_3_O_3_: C, 68.64; H, 6.86; N, 11.44. Found: C, 68.63; H, 6.88; N, 11.45.

### 3.3. Antifungal Activity

#### 3.3.1. Microorganisms and Media

For the antifungal evaluation, standardized strains from the American Type Culture Collection (ATCC, Manassas, VA, USA), and CEREMIC (CCC, Centro de Referencia en Micología, Facultad de Ciencias Bioquímicas y Farmacéuticas, Rosario, Argentina) were used: *C. albicans* ATCC 10231, *S. cerevisiae* ATCC 9763, *C. neoformans* ATCC 32264, *A. flavus* ATCC 9170, *A. fumigatus* ATTC 26934, *A. niger* ATCC 9029, *T. rubrum* CCC 110, *T. mentagrophytes* ATCC 9972, and *M. gypseum* CCC 115. Clinical isolates of *C. neoformans* were provided by Malbrán Institute (IM, Buenos Aires, Argentine). They included five strains of *C. neoformans* whose voucher specimens are presented in Table 3. Strains were grown on Sabouraud-chloramphenicol agar slants for 48 h at 30 °C, were maintained on slopes of Sabouraud-dextrose agar (SDA, Oxoid, Cambridge, UK) and sub-cultured every 15 days to prevent pleomorphic transformations. Inocula were obtained according to reported procedures [29,30] and adjusted to 1–5 × 10^3^ cells with colony-forming units (CFU)/mL.

#### 3.3.2. Antifungal Susceptibility Testing

Minimum Inhibitory Concentration (MIC) of each compound was determined by using broth microdilution techniques according to the guidelines of the Clinical and Laboratory Standards Institute for yeasts (M27-A3) [29] and for filamentous fungi (including dermatophytes) (M38 A2) [30]. MIC values were determined in RPMI-1640 (Sigma-Aldrich) buffered to pH 7.0 with MOPS. Microtiter trays were incubated at 35 °C for yeasts and *Aspergillus* spp. and at 28–30 °C for dermatophyte strains in a moist, dark chamber, and MICs were visually recorded at 48 h for yeasts, and at a time according to the control fungus growth, for the rest of fungi. For the assay, stock solutions of pure compounds were two-fold diluted with RPMI from 250 to 0.98 µg/mL (final volume = 100 µL) and a final DMSO concentration ≤1%. A volume of 100 µL of inoculum suspension was added to each well with the exception of the sterility control where sterile water was added to the well instead. Terbinafine (Novartis Co., Basel, Switzerland) and amphotericin B (Sigma-Aldrich) were used as positive controls. Endpoints were defined as the lowest concentration of drug resulting in total inhibition (MIC_100_) of visual growth compared to the growth in the control wells containing no antifungal drug.

#### 3.3.3. Fungal Growth Inhibition Percentage Determination

This second order test was performed with the yeast *C. neoformans* ATCC 32264 by using the Reference Method for Broth Dilution Antifungal Susceptibility Testing of Yeasts, Approved Standard M27-A3 was used [29]. For the assay, compound test wells (CTWs) were prepared with stock solutions of each compound in DMSO (maximum concentration ≤ 1%), diluted with RPMI-1640, to final concentrations of 250–0.98 μg/mL. An inoculum suspension (100 μL) was added to each well (final volume in the well = 200 μL). A growth control well (GCW) (containing medium, inoculum, and the same amount of DMSO used in a CTW, but compound-free) and a sterility control well (SCW) (sample, medium, and sterile water instead of inoculum) were included for each fungus tested. Microtiter trays were incubated in a moist, dark chamber at 30 °C for 48 h for both yeasts. Microplates were read in a Versa Max microplate reader (Molecular Devices, Sunnyvale, CA, USA). Amphotericin B was used as positive control. Tests were performed in triplicate. Reduction of growth for each compound concentration was calculated as follows: % of inhibition = 100 − (OD_405_ CTW − OD_405_ SCW)/(OD_405_ GCW − OD_405_ SCW). The means ± SEM were used for constructing the dose–response curves representing % inhibition vs. concentration of each compound.

### 3.4. Computational Details

Initially, atomic coordinates for all 14 synthesized compounds and the three inhibitors of chitin synthase 2 (linderone, methyllinderone and kanakugiol) were built using the Avogadro software [47]. Subsequently, a fast geometry relaxation in vacuum was performed with the HF-3c method [48] implemented in Orca 3.0 [49] and then, a most exhaustive geometry optimization and frequency calculations were performed with the *ω*B97X-D functional and the 6-31G(d,p) basis set in Gaussian 09 [50]. For linderone, calculations were performed for both the keto and enol forms and the further analysis were done using the lowest energy isomer, which was the enol form. Next, the energy for the highest occupied molecular orbital (*E_HOMO_*) and the energy for lowest unoccupied molecular orbital (*E_LUMO_*) were obtained directly from these DFT calculations. Global reactivity descriptors as chemical hardness (*η*), electronegativity (*χ*), electronic chemical potential (*µ*), chemical softness (*S*) and the electrophilicity index (*ω*) were obtained from the ionization potential (*I*) and the electron affinity (*A*) of the molecules following the Koopmans’ theorem [39] as:

(1) $$I\approx -E_{HOMO}\;\mathrm{and}\;A\approx -E_{LUMO}$$

(2) $$\eta \approx \left(I-A\right)\approx \left(E_{LUMO}-E_{HOMO}\right)$$

(3) $$\chi =-\mu \approx \frac{1}{2}\left(I+A\right)\approx -\frac{1}{2}\left(E_{HOMO}+E_{LUMO}\right)$$

(4) $$S=\frac{1}{\eta }$$

(5) $$\omega =\frac{\mu^{2}}{2\eta }$$

Condensed-to-atoms Fukui functions (*f^α^_k_*, *α* = +, − or 0) at the *k*th atom were computed using the frontier molecular orbital (FMO) method implemented in the UCA-FUKUI software [51] as follows:

(6) $$f_{k}^{-}=\underset{v\in k}{\sum }\left[{\left|C_{vH}\right|}^{2}+C_{\nu H}\underset{\chi \neq \nu }{\sum }C_{\chi H}S_{\chi \nu }\right]\left(\mathrm{electrophilic}\;\mathrm{attack}\right)$$

(7) $$f_{k}^{+}=\underset{v\in k}{\sum }\left[{\left|C_{vL}\right|}^{2}+C_{\nu L}\underset{\chi \neq \nu }{\sum }C_{\chi L}S_{\chi \nu }\right]\left(\mathrm{nucleophilic}\;\mathrm{attack}\right)$$

(8) $$f_{k}^{0}=\frac{1}{2}\left(f_{k}^{+}+f_{k}^{-}\right)\left(\mathrm{radicalary}\;\mathrm{attack}\right)$$

where *C_νH_* and *C_νL_* are the HOMO and LUMO frontier orbital coefficients, respectively, while *S_χν_* are the atomic orbital overlap matrix elements. After, local softness (*s^α^_k_*) and philicity indexes (*ω^α^_k_*) were computed from Fukui functions (Equations (6)–(8)) and global reactivity parameters (Equations (4) and (5)) as:

(9) $$s_{k}^{\alpha }=Sf_{k}^{\alpha }\left(\alpha =+,-\;or\;0\right)$$

(10) $$\omega_{k}^{\alpha }=\omega f_{k}^{\alpha }\left(\alpha =+,-\;or\;0\right)$$

## 4. Conclusions

In summary, we report here the synthesis of a novel series of (*E*)-1-aryl-3-(4-(2-(dimethylamino)ethoxy)-3-methoxyphenyl)prop-2-en-1-ones **4** and their 3-aryl-5-(4-(2-(dimethylamino)ethoxy)-3-methoxyphenyl)-4,5-dihydro-1*H*-pyrazole-1-carbaldehyde derivatives **5**. Analysis of their antifungal activity allowed us to determine that the vanillin chalcones **4** present better activity than the vanillin pyrazolines **5** and, among the chalcones **4a**–**g**, compound **4a** showed the best antifungal activities, mainly against the dermatophytes *T. rubrum*, *T. mentagrophytes* and *M. gypseum* and the clinically important yeast *C. neoformans*. The most active compound was the chalcone **4a**, which possesses a 4-Cl substituent on ring A, followed by those that have a 4-F (**4b**), a 4-CH_3_ (**4c**) and the non-substituted **4g**. Finally, a chemical reactivity analysis from DFT calculations demonstrated that the higher antifungal activity of chalcones **4** in comparison to pyrazolines **5** is mainly due to the higher electrophilic character of the former compared to the latter. Also, chalcone derivatives with electron-withdrawing substituents on ring A showed higher electrophilicity and subsequently, higher antifungal activity in comparison with electron-donating substituted chalcones.

## Figures and Tables

**Table 1:** Minimum inhibitory concentration and minimum fungicidal concentration of compounds **4a**–**g** and **5a**–**g** (MIC/MFC in µg/mL).

Compound	Structure	Fungal Species							
		*C.a.*	*C.n.*	*A.fu.*	*A.fl.*	*A.n.*	*M.g.*	*T.r.*	*T.m.*
**4a**	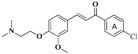	125/250	62.5/125	i	i	i	31.25/31.25	31.25/31.25	31.25/31.25
**4b**	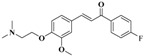	250/>250	125/125	i	i	i	62.5/125	62.5/125	62.5/125
**4c**	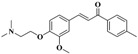	250/nf	125/125	i	i	i	62.5/62.5	62.5/62.5	31.25/31.25
**4d**	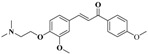	i	250/250	i	i	i	62.5/250	62.5/250	62.5/125
**4e**	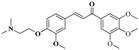	i	i	i	i	i	i	i	i
**4f**	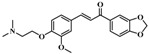	i	250/nf	i	i	i	250/250	250/250	125/250
**4g**	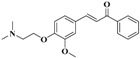	i	125/250	i	i	i	62.5/250	62.5/125	62.5/125
**5a**	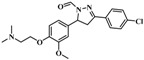	i	125/125	i	i	i	125/250	125/250	125/250
**5b**	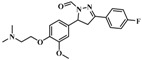	i	250/250	i	i	i	i	i	i
**5c**	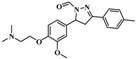	i	250/nf	i	i	i	250/nf	250/nf	250/nf
**5d**	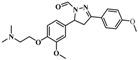	i	i	i	i	i	i	i	i
**5e**	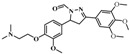	i	i	i	i	i	i	i	i
**5f**	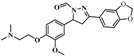	i	i	i	i	i	i	i	i
**5g**	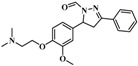	i	i	i	i	i	i	i	i
**Amphotericin B**	-	0.78	0.25	0.50	0.50	0.50	0.12	0.07	0.07
**Terbinafine**	-	0.50	0.25	0.12	0.50	0.25	0.05	0.02	0.02

Antifungal activity was determined with the microbroth dilution assay following the CLSI guidelines; i = MIC > 250 µg/mL; nf: not fungicide up to 250 µg/mL; *C.a.*: *Candida albicans* ATCC 10231; *C.n.*: *Cryptococcus neoformans* ATCC 32264; *A.n.*: *Aspergillus niger* ATCC 9029; *A.fl.*: *Aspergillus flavus* ATCC 9170; *A.fu.*: *Aspergillus fumigatus* ATCC 26934; *M.g.*: *Microsporum gypseum* CCC 115; *T.r*: *Trichophyton rubrum* CCC 110; *T.m.*: *Trichophyton mentagrophytes* ATCC 9972.

**Table 2:**
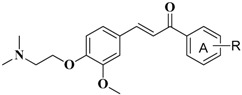
Percentages of inhibition of *C. neoformans* ATCC 32264 by compounds **4a**–**g**.

Concentrations in µg/mL								
R	Compound	250	125	62.5	31.25	15.62	7.81	3.9
4-Cl	**4a**	100	100	96.0 ± 2.0	11.7 ± 2.2	11.2 ± 1.6	0	0
4-F	**4b**	100	100	39.4 ± 2.4	24.5 ± 0.1	14.6 ± 1.7	4.3 ± 0.1	0
4-CH_3_	**4c**	100	93.3 ± 0.7	40.5 ± 0.6	15.5 ± 0.9	0	0	0
4-OCH_3_	**4d**	100	62.4 ± 5.2	26.8 ± 1.4	24.3 ± 0.2	22.6 ± 2.5	16.8 ± 0.4	12.7 ± 3.9
3,4,5-(OCH_3_)_3_	**4e**	30.1 ± 3.1	8.35 ± 1.8	7.6 ± 1.6	4.3 ± 1.7	0	0	0
OCH_2_O	**4f**	100	44.3 ± 4.5	25.8 ± 2.4	18.7 ± 0.1	11.4 ± 3.4	10.4 ± 2.9	7.7 ± 3.1
H	**4g**	100	93.8 ± 9.8	39.6 ± 0.4	26.2 ± 1.7	19.5 ± 7.1	19.3 ± 0.9	11.5 ± 4.8

**Table 3:** MIC_100_, MIC_80_ and MIC_50_ values in µg/mL of compounds **4a**–**c** and **4g** against clinical isolates of *C. neoformans*.

Compound	Clinical Strains of *C. neoformans*																	
	*C.n.* ATCC 32264			*C.n.* IM 983040			*C.n.* IM 972724			*C.n.* IM 042074			*C.n.* IM 983036			*C.n.* IM 00319		
	**MIC_100_**	**MIC_80_**	**MIC_50_**	**MIC_100_**	**MIC_80_**	**MIC_50_**	**MIC_100_**	**MIC_80_**	**MIC_50_**	**MIC_100_**	**MIC_80_**	**MIC_50_**	**MIC_100_**	**MIC_80_**	**MIC_50_**	**MIC_100_**	**MIC_80_**	**MIC_50_**
**4a**	62.5	62.5	62.5	62.5	62.5	62.5	62.5	62.5	62.5	62.5	62.5	62.5	62.5	62.5	62.5	62.5	62.5	62.5
**4b**	125	125	125	125	125	125	125	125	125	125	125	125	125	125	125	125	125	125
**4c**	125	125	62.5	125	125	62.5	125	125	62.5	125	125	62.5	125	125	62.5	125	125	62.5
**4g**	125	125	125	125	125	125	125	125	125	125	125	125	125	125	125	125	125	125
**AmpB**	0.5			0.25			0.25			0.12			0.25			0.5		

*C.n.: Cryptococcus neoformans*.

**Table 4:** Global reactivity indexes for compounds **4a**–**g**, **5a**–**g** and inhibitors of chitin synthase 2 from *S. cerevisiae*.

Compound	*^a^ E_HOMO_*	*^a^ E_LUMO_*	*^a^ η*	*^b^ S* [×10^−4^]	*^a^ χ*	*^a^ ω*
**4a**	−181.43	−9.81	171.62	58.27	95.62	26.64
**4b**	−184.91	−6.80	178.12	56.14	95.86	25.79
**4c**	−182.83	−4.06	178.77	55.94	93.45	24.42
**4d**	−181.33	−2.22	179.12	55.83	91.77	23.51
**4e**	−177.92	−4.74	173.18	57.74	91.33	24.08
**4f**	−177.39	−3.93	173.46	57.65	90.66	23.69
**4g**	−183.73	−5.16	178.56	56.00	94.45	24.98
**5a**	−179.82	5.72	185.54	53.90	87.05	20.42
**5b**	−177.46	11.23	188.69	53.00	83.11	18.30
**5c**	−175.37	13.29	188.66	53.01	81.04	17.41
**5d**	−170.17	16.60	186.77	53.54	76.79	15.78
**5e**	−172.30	14.28	186.58	53.60	79.01	16.73
**5f**	−170.49	13.86	184.35	54.24	78.31	16.63
**5g**	−173.32	12.88	186.20	53.71	80.22	17.28
**Methyllinderone**	−171.65	−2.95	168.70	59.28	87.30	22.59
**Kanakugiol**	−175.60	−8.42	167.18	59.82	92.01	25.32
**Linderone**	−177.74	−11.09	166.65	60.01	94.42	26.75

*^a^* Values in kcal/mol; *^b^* Values in mol/kcal.

**Table 5:** Correlation matrix of global reactivity indexes and pMICs against five fungal species for compounds **4a**–**g**.

	*E_HOMO_*	*E_LUMO_*	*η*	*S*	*χ*	*ω*	*pMIC-C.a.*	*pMIC-C.n.*	*pMIC-M.g.*	*pMIC-T.r.*	*pMIC-T.m.*
***E_HOMO_***	1.00										
***E_LUMO_***	0.26	1.00									
***η***	−0.68	0.53	1.00								
***S***	0.68	−0.53	−1.00	1.00							
***χ***	−0.83	−0.76	0.15	−0.15	1.00						
***ω***	−0.54	−0.96	−0.26	0.26	0.92	1.00					
**pMIC-*C.a.***	−0.01	−0.82	−0.62	0.62	0.49	0.73	1.00				
**pMIC-*C.n.***	−0.59	−0.85	−0.13	0.14	0.89	0.92	0.75	1.00			
**pMIC-*M.g.***	−0.77	−0.49	0.31	−0.30	0.81	0.68	0.56	0.81	1.00		
**pMIC-*T.r.***	−0.77	−0.49	0.31	−0.30	0.81	0.68	0.56	0.81	1.00	1.00	
**pMIC-*T.m.***	−0.68	−0.36	0.32	−0.32	0.67	0.53	0.47	0.77	0.90	0.90	1.00
